# Effects of cinnamon, rosemary and oregano on growth performance, blood biochemistry, liver enzyme activities, excreta microbiota and ileal morphology of *Campylobacter jejuni*‐challenged broiler chickens

**DOI:** 10.1002/vms3.70034

**Published:** 2024-09-18

**Authors:** Zahra Alimohammadi, Hassan Shirzadi, Kamran Taherpour, Enayat Rahmatnejad, Ali Khatibjoo

**Affiliations:** ^1^ Department of Animal Science Faculty of Agriculture Ilam University Ilam Iran; ^2^ Department of Animal Science Faculty of Agriculture and Natural Resources Persian Gulf University Bushehr Iran

**Keywords:** blood profile, broiler chickens, *Campylobacter jejuni*, ileal morphology, performance, phytogenic additives

## Abstract

**Background:**

Phytogenic additives would be helpful to mitigate the detrimental impact of *Campylobacter jejuni* on broiler chickens.

**Objective:**

The experiment aimed to assess the effects of cinnamon, rosemary and oregano powder on physiological responses of broiler chickens challenged with *C. jejuni* from 0 to 42 days of age.

**Methods:**

A total of 192 one‐day‐old male broiler chickens were divided into 6 treatment groups. The treatments included: negative control (NC; basal diet without additives and no *C. jejuni* challenge), positive control (PC; basal diet with *C. jejuni* challenge), PC with cinnamon, rosemary or oregano powder (3 g/kg each), and PC with *Erythromycin* (55 mg/kg). Except for the NC group, all chicks were orally challenged with 2 × 10^8^ CFU/mL *C. jejuni* daily from days 21–25. Feed intake, body weight gain (BWG), feed conversion ratio (FCR), energy efficiency ratio (EER) and protein efficiency ratio (PER) were assessed during the rearing period (0–42 days). On day 42 of age, fresh excreta samples were collected from each pen to determine apparent dry matter digestibility and excreta microbiota. In addition, at the end of the experiment, blood samples were collected to evaluate blood profile and liver enzyme activities.

**Results:**

*C. jejuni* challenge (PC treatment) decreased BWG, EER and PER, while increasing FCR of broiler chickens (*p* < 0.05), whereas rosemary, oregano and *Erythromycin* improved these performance parameters akin to NC. PC diet showed negative effect in ileal morphology, alleviated by additives except cinnamon (*p* < 0.05). Dietary additives successfully reduced *Campylobacter* levels and increased *Lactobacilli* counts in the PC. Rosemary and oregano lowered plasma total cholesterol (*p* < 0.05). Alanine aminotransferase elevation by *C. jejuni* challenge in the PC group was prevented by rosemary, oregano and *Erythromycin* (*p* < 0.05).

**Conclusions:**

Oregano and rosemary alleviate the impact of *C. jejuni* challenge.

## Graphical abstract

• The purpose of the study was to evaluate the effects of cinnamon, rosemary and oregano powder on the physiological responses of broiler chickens challenged with *Campylobacter jejuni*.

• *C. jejuni* challenge decreased body weight gain, energy and protein efficiency ratios, and feed efficiency of broiler chickens.

• Oregano and rosemary mitigate the negative effect of *C. jejuni* on the broiler production.

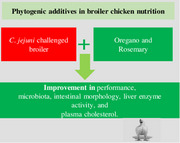

## 1 INTRODUCTION

Feed constitutes a fundamental element in the poultry industry, significantly influencing the gastrointestinal tract and its overall health. The gastrointestinal tract is recognized as a sophisticated and dynamic organ critical for gut health. Numerous factors, including stressors, can potentially disturb the delicate balance of the gut ecosystem, leading to adverse effects on poultry health and productivity. Gastrointestinal dysbiosis poses significant challenges to the poultry sector by compromising gut health and elevating disease vulnerability. In contrast, a healthy gut in poultry facilitates efficient nutrient digestion and absorption, particularly in environments where these stressors are minimized (Diaz Carrasco et al., 2019).

*Campylobacter jejuni*, a gram‐negative, non‐spore‐forming bacterium, is a widespread cause of gastroenteritis (Epps et al., 2013; Kaakoush et al., 2015; Moore et al., 2005). It is a significant pathogen responsible for human diarrheal disease. Poultry serves as a significant source of *C. jejuni* exposure, given that this bacterium thrives within the gastrointestinal tract, posing a major risk for foodborne illnesses in humans worldwide (Beier et al., 2019; Connerton et al., 2018). Its ability to colonize and suppress immune responses in chickens impacts broiler performance (Nooreh et al., 2021).

In the past, antibiotics were employed as growth promoters to combat gastrointestinal pathogens and mitigate the impact of stressors on gut function. Nevertheless, restrictions on antibiotic use in poultry production have been implemented due to rising consumer awareness about antibiotic‐related health risks, bacterial resistance and food safety concerns (Ali et al., 2021). In light of the constraints imposed on antibiotic use in chicken production in recent years, the poultry industry has turned to alternative approaches, such as incorporating plant‐derived feed additives. The rising interest in plant bioactive compounds, such as phytochemicals, within poultry diets stems from their promising capabilities as antioxidants and antimicrobial agents. As a result, these natural compounds are increasingly sought‐after for their potential to support the well‐being and health of poultry while aligning with the trend of reducing antibiotic usage (Ali et al., 2021).

Despite some reports on phytogenic additives as antibiotic alternatives for *Campylobacter*, only a few have been thoroughly studied. Inconsistent results emphasize the need for further research to understand their potential in poultry diets. Cinnamon, from the genus *Cinnamomum* (Lauraceae family), comprises over 250–300 aromatic evergreen shrubs and plant trees. However, only a few of these species hold substantial economic importance as a widely used spice globally (Ali et al., 2021). Cinnamon is rich in bioactive compounds, including volatile oils, flavonoids, curcuminoids, coumarins, tannins, alkaloids, xanthones, terpenoids, phenolics and more. These components contribute to its natural antioxidant, antimicrobial and anti‐inflammatory properties (Liyanage et al., 2021; Ribeiro‐Santos et al., 2017). Rosemary (*Rosmarinus officinalis*) is recognized for its medical benefits and aromatic properties, making it popular for various applications (Belenli et al., 2015). As a natural animal feed additive, its antimicrobial activity and antioxidant properties play a significant role (Abd El‐Latif et al., 2013). These properties are attributed to the presence of bioactive compounds, including 1,8‐cineole, rosemarinic acid and rosmarole, making it a valuable source of antioxidative compounds (Abd El‐Latif et al., 2013; Franciosini et al., 2016; Jiang et al., 2011). Oregano (*Origanum vulgare* L.) is a highly aromatic herb with abundant active chemical compounds (Park et al., 2015). Oregano essential oil contains major components such as carvacrol and thymol, terpenoid compounds with antimicrobial properties (Teixeira et al., 2013). The polyphenols in oregano have also been studied for their antimicrobial activity and molecular mechanisms in suppressing microbes (Xu et al., 2008). Additionally, oregano extract serves as a natural antioxidant, effectively preventing lipid oxidation and contributing to improved meat quality and animal health (Lee et al., 2004).

Several studies have reported that these medicinal plants positively influence the performance, intestinal morphology, nutrient digestibility and gut microbiota of poultry (Abd El‐Latif et al., 2013; Ashayerizadeh et al., 2009; Belenli et al., 2015; Franciosini et al., 2016; Mathlouthi et al., 2012; Ri et al., 2017; Saki et al., 2012). For example, the results of a study demonstrated that supplementing cinnamon led to significant improvements in body weight gain (BWG) and feed conversion ratio (FCR) in broilers, comparable to the group fed with oxytetracycline antibiotic (Singh et al., 2014). Moreover, Kanani et al. (2016) reported that including of 0.5% cinnamon in the feed significantly increased both feed intake (FI) and BWG compared to the control birds. Moreover, the use of rosemary in broiler chickens diets increased average daily gain, feed efficiency and European production efficiency factor (Petricevic et al., 2018). In another study, Ri et al. (2017) reported that the oregano supplementation increased broiler chickens' daily gain and daily FI during the grower period and the whole period.

Indeed, most of the previous research has primarily focused on investigating the effects of medicinal plants on poultry with normal health conditions. Therefore, this study sought to explore how cinnamon, rosemary and oregano powder affect broiler chickens challenged with *C. jejuni* regarding growth performance, excreta microbiota, intestinal morphology and nutrient digestibility.

## 2 MATERIALS AND METHODS

### 2.1 Experimental design and diets

A total of 192 1‐day‐old male broiler chicks (Ross 308) were sourced from a commercial hatchery. Using a completely randomized design, the chicks were weighed and randomly assigned to 24 pens with 6 treatments and 4 replicate groups of 8 birds each. The trial began at 32°C room temperature for the first 3 days, gradually decreasing by 3°C weekly until reaching 20°C. Cinnamon, rosemary and oregano plants (from herbal store, Ilam, Iran) were uses as powder in broiler diets. The essential oils composition of plants was determined using gas chromatography (GC; Agilent 6890N, Agilent Technologies) interfaced with mass spectroscopy (Agilent 5973N, Agilent Technologies). Cinnamon oil contained high levels of cinnamaldehyde (77.28%) and camphor (6.12%), rosemary oil had elevated levels of 1,8‐cineole (33.4%) and camphor (12.48%), whereas oregano oil exhibited significant concentrations of carvacrol (57.86%) and thymol (18.87%). The experimental treatments were as follow: negative control (NC; basal diet without additive and without *C. jejuni* challenge), positive control (PC; basal diet without additives but challenged with *C. jejuni*), PC + cinnamon powder (3 g/kg), PC + rosemary powder (3 g/kg), PC + oregano powder (3 g/kg) and PC + *Erythromycin* (55 mg/kg). This study employed a two‐phase feeding programme consisting of starter diets for 0–21 days and finisher diets for 22–42 days. Experimental diets were formulated according to the nutritional requirements of broiler chickens described in NRC (1994). Table 1 presents the feed components and chemical composition of the basal diet. Except for the NC group, chicks were orally challenged with 2 × 10^8^ CFU/mL *C. jejuni* (1 mL/bird) from days 21 to 25 of age.

**TABLE 1:** Ingredients and nutrient contents of basal diets (g/kg, unless otherwise stated).

	Starter (0–21 days)	Grower (22–42 days)
**Ingredients**		
Corn grain	647.2	699.8
Soybean meal (44% CP)	238.7	234.8
Corn gluten meal	59.30	−
Corn oil	9.100	23.20
l‐Lysine‐HCl	2.000	2.000
dl‐Methionine	1.700	1.700
l‐Threonine	0.300	1.100
Dicalcium phosphate	17.30	12.90
Limestone	11.60	12.60
Common salt	3.400	2.200
Sodium bicarbonate	1.400	1.700
Sand^a^	3.000	3.000
Mineral and vitamin premix^b^	5.000	5.000
**Calculated composition**		
Metabolizable energy (Kcal/kg)	3000	3100
Crude protein	200.0	176.6
Calcium	9.400	8.700
Available phosphorus	4.200	3.400
Sodium	1.900	1.500
Lysine	10.30	9.700
Arginine	11.70	10.70
Threonine	7.500	7.200
Methionine + cystine	8.400	7.000

^a^ The sand was replaced (wt/wt) by cinnamon, rosemary, oregano or *Erythromycin* to form the corresponding experimental diets.

^b^ Supplied as retinyl acetate, 9000 IU; cholecalciferol, 2000 IU; dl‐α‐tocopheryl acetate, 12.5 IU; menadione sodium bisulphite, 1.76 mg; choline chloride, 320 mg; nicotinic acid, 28 mg; calcium d‐pantothenate, 6.4 mg; riboflavin, 3.2 mg; pyridoxine, 1.97 mg; thiamine, 1.2 mg; folic acid, 0.38 mg; biotin, 0.12 mg; cyanocobalamin, 0.01 mg; FeSO_4_·7H_2_O, 80 mg; MnSO_4_·H_2_O, 60 mg; ZnO, 51.74 mg; CuSO_4_·5H2O, 8 mg; iodized NaCl, 0.8 mg; Na_2_SeO_3_, 0.2 mg/kg of diet.

### 2.2 Growth performance and digestibility

FI was calculated as the difference between the offered and refused feed during the starter (0–21 days) and the grower (22–42 days). BWG was recorded, and FCR was determined as FI divided by BWG.

Moreover, the growth efficiency parameters, including the energy efficiency ratio (EER) and protein efficiency ratio (PER), were computed using the following formulas (Mir et al., 2018):

EER = [Weight gain (g)/metabolizable energy intake (kcal)] × 100
PER = Weight gain (g)/crude protein intake (g)

To assess dry matter (DM) digestibility, all diets contained 3 g/kg of chromium oxide (Cr_2_O_3_), an inert marker, from days 39 to 42 of age. On day 42 of age, fresh excreta samples were collected from each pen to determine apparent DM digestibility. DM content of diets and excreta were determined using the method outlined in AOAC (2005). Excreta samples were stored at −20°C until analysis. Subsequently, chromium was determined based on the method proposed by Shi et al. (2022). In summary, the samples were ashed in a muffle furnace at 600°C for 24 h and digested in citric acid. The mixture was slowly heated for 40 min and then incubated in HClO_4_ for 3 h at 140°C. Finally, the absorbance at 405 nm was measured using an atomic absorption spectrometer (AA 670; Shimadzu). Furthermore, the apparent DM digestibility coefficients were calculated using the following equation (Rehman et al., 2016):

Apparent DM digestibility = 100 − [(% marker in diet/% marker in excreta) × 100]

### 2.3 Ileal morphology

Histomorphology study involved selecting two birds with body weights close to the average weight of each pen's replicate and slaughtering them by cervical dislocation at the trial's end. Ileal tissue samples were fixed in 10% neutral buffered formalin for 24 h, followed by dehydration, clearing and paraffin embedding. Five‐micrometre sections were cut using a rotary microtome, mounted on glass slides and stained with alcian blue, periodic acid Schiff, haematoxylin and eosin. The villus height (VH) was defined as the distance from the villus tip above the lamina propria, whereas the villus width (VW) was measured at the basal and apical ends, and the mean widths were calculated. The villous surface area (VSA) and VH to crypt depth (CD) ratio were calculated according to Baurhoo et al. (2011). All measurements were conducted using a computer‐aided light microscope image analysis.

### 2.4 Excreta microbiology

At 42 days of age, fresh excreta samples were collected and taken to the microbial lab on ice. According to Serpunja and Kim (2019), samples were diluted in phosphate‐buffered saline, and serial dilutions (10^−3^–10^−7^) were prepared. Then, five 20 µL volumes of each dilution were plated on separate media for analysis. Plate count agar (Cat No. QB‐65‐5240, Quelab) media, *Campylobacter* selective agar (Cat No. 1.02249, Merck), brilliance phenol red green agar (Cat No. QB‐65‐5236, Quelab), *Bifidobacterium* selective agar (Cat No. B1143, Biomark) and the de Man, Rogosa and Sharpe agar (Cat No. QB‐65‐5262, Quelab) media were used for isolation of total aerobic bacteria, *C. jejuni*, *Salmonella, Bifidobacterium* and *Lactobacillus*, respectively. Bacterial counting occurred after aerobic chamber incubation at 37°C for 24 h, followed by logarithmic (log_10_) transformation of microbial colony‐forming units.

### 2.5 Blood profile, atherogenic indices and liver enzyme activities

At the end of the experiment, blood samples from four birds in each treatment were collected in non‐heparinised tubes from individual birds followed by serum harvesting and storage at −20°C until analysis. The serum glucose, triglyceride, total cholesterol, high‐density lipoprotein cholesterol (HDL), low‐density lipoprotein cholesterol (LDL), aspartate aminotransferase (AST), alanine aminotransferase (ALT), alkaline phosphatase (ALP) and lactate dehydrogenase (LDH) activities were assessed using specific kits (Pars Azmoun) and a spectrophotometer (BT1500, Biotechnica). Moreover, the serum atherogenic indices, including the cardiac risk ratio (CRR), atherogenic coefficient (AC) and atherogenic index (AI), were determined following the formulas outlined by (Dev et al., 2020)

CRR = Total cholesterol/HDL cholesterol
AC = (Total cholesterol − HDL cholesterol)/HDL cholesterol
AI = log (Triglycerides/HDL cholesterol)

### 2.6 Statistical analysis

Statistical analyses were performed using the SAS software package (SAS Institute Inc., 2010). The pen was the experimental unit, and a completely randomized design with the GLM procedure was employed. The Kolmogorov–Smirnov and Levene tests were used to test normality and variances. Tukey's multiple tests compared significant differences (*p* ≤ 0.05).

## 3 RESULTS

### 3.1 Growth performance and digestibility

Table 2 illustrates the impact of dietary treatments on the growth performance and DM digestibility of broiler chickens exposed to *C. jejuni* challenge. The introduction of the *C. jejuni* challenge in the PC group negatively affected bird performance, leading to decreased BWG, EER and PER, along with an increase in FCR (*p* < 0.05). The inclusion of rosemary, oregano and *Erythromycin* alleviated this decline in performance (*p* < 0.05), resembling the outcomes observed in the NC group. Regarding BWG, EER, PER and FCR, it is worth noting that the treatment with cinnamon showed comparatively less effectiveness than the other additives. This indicates that although cinnamon improved growth performance parameters, there was no significant difference compared to the NC and PC groups. Additionally, there were no significant differences in FI, excreta DM and DM digestibility among the experimental treatments (*p* > 0.05). *C. jejuni*‐challenged broilers (PC group) exhibited lower values for excreta DM and DM digestibility compared to the other treatment groups.

**TABLE 2:** Effects of dietary treatments on growth performance and dry matter digestibility in broiler chickens challenged with *Campylobacter jejuni*.

	NC	PC	Cinnamon	Rosemary	Oregano	*Erythromycin*	SEM	*p*‐value
BWG	2481^a^	2259^b^	2404^ab^	2523^a^	2569^a^	2538^a^	38.2	0.01
FI	4482	4582	4590	4583	4597	4599	56.7	0.69
FCR	1.80^b^	2.03^a^	1.91^ab^	1.82^b^	1.79^b^	1.81^b^	0.033	0.01
EER	18.0^a^	16.1^b^	17.0^ab^	17.9^a^	18.2^a^	17.9^a^	0.3	<0.01
PER	3.17^a^	2.82^b^	3.00^ab^	3.15^a^	3.20^a^	3.16^a^	0.06	<0.01
DM	17.0	15.4	17.3	17.8	16.7	18.0	0.8	0.32
DM_dig_	73.1	70.1	71.3	74.4	75.7	74.4	1.4	0.09

*Note*: Means within a row sharing a common superscript (a, b) are not different (*p* > 0.05).

Abbreviations: BWG, body weight gain (g); DM, dry matter (%); DM_dig_, dry matter digestibility (%); EER, metabolisable energy efficiency ratio (%); FCR, feed conversion ratio (g/g); FI, feed intake (g); NC, negative control, basal diet without additive and without of *C. jejuni* challenge; PC, positive control, basal diet without additives but challenged with *C. jejuni*; PER, protein efficiency ratio (g/g); SGR, specific growth rate (% per day).

### 3.2 Ileal morphology

Table 3 shows the influence of dietary treatments on the ileal morphology of broiler chickens exposed to *C. jejuni* challenge. The broilers fed with the PC diet demonstrated a decrease (*p* < 0.05) in VH, VH to CD ratio and VSA, yet displayed no adverse impact on VW and CD (*p* > 0.05). Incorporating additives mitigated this detrimental impact on ileal morphology, mirroring the results seen in the NC group (*p* < 0.05). It is important to highlight that the cinnamon‐containing treatment displayed relatively less effectiveness compared to other additives, as cinnamon solely enhanced the VH to CD ratio in comparison to the PC group.

**TABLE 3:** Effects of dietary treatments on ileal morphology of broiler chickens challenged with *Campylobacter jejuni*.

	NC	PC	Cinnamon	Rosemary	Oregano	*Erythromycin*	SEM	*p*‐value
VH	872^a^	801^b^	842^ab^	847^ab^	887^a^	880^a^	12	<0.01
VW	150	145	150	151	149	151	2	0.12
CD	138	143	139	137	138	136	2	0.14
VH:CD	6.32^ab^	5.61^c^	6.06^b^	6.19^ab^	6.43^ab^	6.49^a^	0.09	<0.01
VSA	0.411^a^	0.364^b^	0.396^ab^	0.403^a^	0.416^a^	0.418^a^	0.007	<0.01

*Note*: Means within a row sharing a common superscripts (a–c) are not different (*p* > 0.05).

Abbreviations: CD, crypt depth; NC, negative control, basal diet without additive and without of *C. jejuni* challenge; PC, positive control, basal diet without additives but challenged with *C. jejuni*; VH, villus height (µm); VSA, villus surface area (mm^2^); VW, villus width (µm).

### 3.3 Excreta microbiota

The effects of different dietary treatments on the excreta microbiota of broilers are presented in Table 4. The experimental treatments did not significantly influence the total counts of aerobic bacteria, *Salmonella* and *Bi* *fidobacteria* (*p* > 0.05). However, the introduction of *C. jejuni* challenge increased *Campylobacter* colonization in birds fed with the PC diet. Remarkably, all treatments incorporating additives exhibited reduced *Campylobacter* colonization compared to the PC group (*p* < 0.05).

**TABLE 4:** Effects of dietary treatments on broiler chickens’ excreta microbial shedding challenged with *Campylobacter jejuni* (log_10_ CFU/g).

	NC	PC	Cinnamon	Rosemary	Oregano	*Erythromycin*	SEM	*p*‐value
Total aerobic bacteria	9.66	10.68	10.29	10.10	9.66	9.98	0.321	0.25
*Campylobacter*	7.55^c^	8.92^a^	8.21^b^	7.86^bc^	7.93^bc^	7.72^bc^	0.141	<0.01
*Salmonella*	7.94	8.42	8.49	8.19	8.36	7.86	0.340	0.71
*Bifidobacteria*	8.78	7.72	8.61	8.62	8.54	8.40	0.371	0.43
*Lactobacillus*	8.72^a^	7.51^bc^	8.32^ab^	8.53^a^	8.87^a^	7.36^c^	0.208	<0.01

*Note*: Means within a row sharing a common superscripts (a–c) are not different (*p* > 0.05).

Abbreviations: NC, negative control, basal diet without additive and without of *C. jejuni* challenge; PC, positive control, basal diet without additives but challenged with *C. jejuni*.

Regarding *Lactobacilli* counts, the *C. jejuni* challenge resulted in a decline in *Lactobacilli* colonization within the PC group. Consequently, this group exhibited lower *Lactobacilli* count than the NC group and others (*p* < 0.05). Nonetheless, treatments incorporating rosemary, oregano and, to a certain extent, cinnamon were effective in preventing the reduction in *Lactobacilli* counts, showcasing effect akin to the NC group (*p* < 0.05). This protective effect, however, was not observed in the group receiving *Erythromycin* (*p* > 0.05).

### 3.4 Blood profile, atherogenic indices and liver enzyme activities

The effects of dietary treatments on the blood profile and atherogenic indices and liver enzyme activities of broilers are presented in Table 5. The results showed that *C. jejuni* challenge has no effect on the plasma concentration of glucose, triglyceride, HDL, LDL and total cholesterol in the PC group (*p* > 0.05). Moreover, the concentration of glucose, triglyceride, HDL and LDL was not affected by the treatments containing additives (*p* > 0.05). Although in terms of total cholesterol concentration, no difference was observed between the treatments containing additives and the NC group; however, rosemary and oregano supplementation led to a decrease in its plasma concentration compared to the PC group (*p* < 0.05). Moreover, none of the plasma atherogenic indices, including CRR, AC and AI, were affected by bacterial challenge or feed additives (*p* < 0.05).

**TABLE 5:** Effects of dietary treatments on blood biochemistry (mg/dL) and atherogenic indices in broiler chickens challenged with *Campylobacter jejuni*.

	NC	PC	Cinnamon	Rosemary	Oregano	*Erythromycin*	SEM	*p*‐value
Glucose	239	250	247	239	234	233	14	0.94
Triglyceride	103	95.5	80.5	99.8	98.0	82.8	10.5	0.57
Total cholesterol	155^abc^	168^a^	161^abc^	140^c^	142^bc^	163^ab^	5	<0.01
HDL	29.5	32.8	35.0	38.5	39.3	36.5	3.0	0.23
LDL	56.0	60.0	48.0	46.8	44.3	49.0	3.6	0.04
CRR	5.40	5.31	4.72	3.77	3.67	4.55	0.47	0.08
AC	4.40	4.31	3.72	2.77	2.67	3.55	0.47	0.08
AI	0.543	0.445	0.365	0.416	0.395	0.356	0.068	0.44

*Note*: Means within a row sharing common superscripts (a–c) are not different (*p* > 0.05).

Abbreviations: AC, atherogenic coefficient; AI, atherogenic index; CRR, cardiac risk ratio; HDL, high‐density lipoprotein; LDL, low‐density lipoprotein; NC, negative control, basal diet without additive and without of *C. jejuni* challenge; PC, positive control, basal diet without additives but challenged with *C. jejuni*.

The impacts of various dietary treatments on the liver enzyme activities of broilers are outlined in Table 6. The administered treatments did not yield significant changes in AST, ALP and LDH levels (*p* > 0.05). The challenge with *C. jejuni* led to an elevation in ALT levels within the PC group (*p* < 0.05). However, diets containing rosemary, oregano, *Erythromycin* and, to some extent, cinnamon effectively averted the rise in ALT, demonstrating a response similar to that of the NC group (*p* < 0.05).

**TABLE 6:** Effects of dietary treatments on liver enzyme activities (U/L) in broiler chickens challenged with *Campylobacter jejuni*.

	NC	PC	Cinnamon	Rosemary	Oregano	*Erythromycin*	SEM	*p*‐value
AST	211	217	210	193	195	206	13.9	0.80
ALT	5.25^bc^	7.50^a^	6.50^ab^	5.75^bc^	4.75^c^	5.25^bc^	0.35	<0.01
ALP	790	816	814	758	803	796	59.1	0.98
LDH	525	513	594	527	508	474	53.1	0.74

*Note*: Means within a row sharing common superscripts (a–c) are not different (*p* > 0.05).

Abbreviations: NC, negative control, basal diet without additive and without of *C. jejuni* challenge; PC, positive control, basal diet without additives but challenged with *C. jejuni*; AST, aspartate aminotransferase; ALT, alanine aminotransferase; ALP, alkaline phosphatase; LDH, lactate dehydrogenase.

## 4 DISCUSSION

The results highlight several noteworthy findings that contribute to our understanding of the complex interactions between dietary treatments and *C. jejuni* challenge in broiler chickens’ production. The results revealed that the introduction of a *C. jejuni* challenge had a detrimental effect on birds’ performance, leading to reduced BWG and elevated FCR. These findings corroborate existing literature that establishes the negative impact of *Campylobacter* colonization on broiler growth and efficiency (Guyard‐Nicodème et al., 2016). Importantly, treatments containing additives displayed a promising ability to mitigate the adverse effects of the *Campylobacter* challenge, resulting in performance resembling that of the NC group. These results emphasize the potential of medicinal plants in supporting broiler performance under pathogenic stressors. Notably, although the cinnamon‐supplemented treatment exhibited relatively lower efficacy compared to other additives, it still demonstrated potential in alleviating the impact of *Campylobacter* challenge.

According to reports, antibiotics are known to enhance animal growth by altering bacterial metabolism (Ghunaim, 2009). Furthermore, exposure to antibiotics creates selective pressure within the digestive systems of animals, reducing the growth and spread of harmful bacterial strains such as *Salmonella* and *Campylobacter* (Ghunaim, 2009). As a result, the observed performance enhancement in the *Erythromycin*‐fed group can be attributed to alterations in pathogen metabolism and reduced colonization. The FCR, representing the relationship between FI and weight gain, became more pronounced due to the decrease in growth rate induced by *C. jejuni* challenge in broilers. As the challenge primarily impacted growth rate without affecting FI, the resulting increase in FCR within the *C. jejuni*‐infected group (PC) is readily apparent. Aligning with the present findings, existing literature highlights that broiler challenge with *C. jejuni* elevates the FCR (Gharib Naseri et al., 2012). However, this unfavourable effect can be mitigated through the incorporation of dietary supplements such as organic acids, probiotics and medicinal plants (Gharib Naseri et al., 2012).

Given that thymol and carvacrol are the principal and most potent constituents of oregano essential oil (Kırkpınar et al., 2011), and these two compounds are attributed to oregano's antimicrobial activity (Mathlouthi et al., 2012), the enhanced performance observed in the oregano‐fed group can be linked to the antimicrobial effects, leading to a reduction in *Campylobacter* colonization. This reduction subsequently curtailed bacterial toxin production, facilitated the restoration of near‐normal intestinal villi growth and improved nutrient digestion, like the characteristics observed in the NC group.

Verbenone, 1,8‐cineole, camphor and borneol have been identified as the key compounds responsible for the antimicrobial properties of rosemary essential oil (Mathlouthi et al., 2012). Consequently, the improved performance observed in the rosemary‐fed group can be attributed to the reduction in *Campylobacter* colonization. Furthermore, a separate study attributed enhanced growth performance in chickens fed with rosemary leaves to increased apparent dietary protein digestibility, along with improved nutrient availability in the intestines for efficient absorption, leading to accelerated growth (Botsoglou et al., 2007). This highlights yet another contributing factor to the improved performance of chickens receiving rosemary supplementation.

The inclusion of cinnamaldehyde in broiler diets has been cited as a promoter of growth performance attributed to its antimicrobial influence against detrimental bacteria and its enhancement of digestibility (Mehdipour et al., 2013). In our current experiment, the incorporation of cinnamon powder demonstrated performance improvement; however, this effect stood between the outcomes of the PC and NC treatments.

The reduction in *Lactobacilli* count within the PC group following the *C. jejuni* challenge could potentially be attributed to increased *Campylobacter* colonization in the digestive tract. This surge in *Campylobacter* colonization might lead to competitive inhibition, subsequently diminishing the presence of beneficial bacteria like *Lactobacilli*, thereby contributing to dysbiosis in the bird's gut. Notably, several *Clostridium perfringens* species have shown sensitivity to carvacrol, cinnamaldehyde, citral and thymol. Utilizing these compounds is considered an effective strategy for managing the colonization of pathogenic bacteria and preserving intestinal health (Ouwehand et al., 2010).

In the current study, the utilization of *Erythromycin*, despite its effectiveness in diminishing *Campylobacter* colonization, coincided with a reduction in *Lactobacilli* counts. This phenomenon can potentially be attributed to the broad‐spectrum nature of the mentioned antibiotic. Beyond its impact on the reduction of gram‐negative bacteria colonization, it also affects the population of gram‐positive bacteria. In contrast, the utilization of phytogenic additives in this study ameliorated the decrease in *Lactobacillus* counts caused by the *Campylobacter* challenge, aligning their numbers more closely with those of the NC group. This beneficial effect of phytogenic additives could be attributed to their antibacterial properties against *Campylobacter* colonization. By curtailing the proliferation of *Campylobacter*, these compounds facilitate the establishment of a more balanced and stable intestinal microbial population, thereby mitigating the adverse consequences of the challenge.

In accordance with the findings of this study, previous research has indicated that incorporating antibiotics into broiler diets enhances growth performance while also leading to an increase in VH and VW and a decrease in CD (Marković et al., 2009). Phytogenic compounds, on the other hand, have been demonstrated to boost mucus production in the stomach and jejunum, signifying their protective role against gastrointestinal pathogens (Manzanilla et al., 2004). Further supporting these observations, the addition of a combination of thymol and carvacrol at 100 and 200 mg/kg, respectively, to broiler diets has been reported to elevate VH in the jejunum, although it showed no impact on VW (Hashemipour et al., 2013). The observed enhancement in intestinal morphology facilitated by the medicinal plants employed in this study can be attributed to their antibacterial compounds. By curbing *Campylobacter* colonization and preventing the decline in *Lactobacilli* count, these compounds contribute to optimal bacterial toxin concentrations. Consequently, they prevent villus damage and excessive CD, thus averting abnormal villus remodelling.

Plant essential oils serve as beneficial additives that enhance nutrient digestibility by elevating the activity of digestive enzymes and promoting the secretion of gastric and pancreatic juices. Their inherent antimicrobial properties contribute to the preservation of intestinal microvilli, consequently bolstering nutrients absorption. This effect is associated with an overall enhancement in digestibility coefficients (Fascina et al., 2012; Hernandez et al., 2004). Notably, an additional study underscores that the improved digestion and absorption in poultry's digestive systems when utilizing medicinal plants is attributed to the impact of their active components on the secretion of digestive enzymes such as proteases, lipase and amylase, coupled with the augmentation of intestinal villi count (Ponte et al., 2008). In this particular experiment, there was not a notable difference observed between the treatments concerning excreta DM and DM digestibility, contrary to what was mentioned in other cases. One potential reason for this disparity could be attributed to the type and quantity of herbal additives utilized in this research.

Biochemical parameters in the blood reflect the state of body organs influenced by various internal and external factors. According to the findings of this study, as well as research conducted by Lee et al. (2004) and Bampidis et al. (2005), which involved the inclusion of carvacrol, thymol and cinnamaldehyde in the diet of broilers, and oregano along with a commercial product containing essential oils in the diet of turkeys, it was revealed that these potent plant substances could potentially have positive effects on reducing blood cholesterol levels. This effect might occur through the inhibition of the activity of the regulatory enzyme responsible for cholesterol production, namely 3‐hydroxy 3‐methylglutaryl coenzyme A reductase. Additional research conducted by Shamlo et al. (2014) demonstrated that incorporating medicinal plants like oregano into the diet of broilers stimulates the proliferation of beneficial bacteria such as *Lactobacillus* and *Bifidobacterium* within the intestine. This proliferation contributes to a reduction in blood cholesterol levels. These specific bacteria disrupt the intestinal‐hepatic cycle of bile acids through deconjugation, consequently diminishing cholesterol absorption by reducing micelle formation. Traesel et al. (2011) supported our findings by indicating that using levels of 50, 100 and 150 mg/kg of a blend of oregano and rosemary essential oils in broiler diets did not affect HDL, triglyceride levels, total protein, albumin, globulin and the albumin‐to‐globulin ratio. However, in contrast to the outcomes of the current study, the same researchers reported that incorporating oregano and rosemary essential oils in the diet of broiler chickens did not significantly impact serum cholesterol levels. This discrepancy might be attributed to the relatively low levels of essential oils used in their research. Additionally, due to the volatile nature of essential oils leading to rapid evaporation, it might be necessary to prepare rations containing essential oils at least every other day to minimize evaporation and potentially observe their positive effects.

The calculation formulas for atherogenic indices indicate that lower values are indicative of hypocholesterolemic and hypolipidemic effects. Although treatments containing herbal additives did exhibit a reduction in atherogenic indices to some degree, their overall impact was not considered significant. The experimental treatments notably influenced total cholesterol levels but did not sufficiently impact the other components within the atherogenic indices formulas, thereby resulting in minimal alteration of these indicators.

The assessment of poultry health relies heavily on analysing serum enzyme activities. Enzymes like ALT and AST, found predominantly in the liver, serve as specific indicators of hepatic cellular damage and inflammation (Selim et al., 2021). LDH isoenzymes, existing in various bird tissues, including liver, muscle, kidney and blood cells, decrease notably during liver diseases (Viveros et al., 2002). ALP, produced in all tissue types, aids in dephosphorylation and functions optimally in alkaline conditions (Selim et al., 2021). The findings from our study revealed that *C. jejuni* resulted in elevated serum ALT levels, suggesting a potential link to increased tissue damage and the disruption of liver functions. This connection is supported by previous reports associating higher levels of these markers with liver damage and inflammation (Selim et al., 2021; Viveros et al., 2002). Our findings align with reports stating that carvacrol usage inhibits blood biochemical changes induced by acrylamide, such as heightened ALT enzyme concentrations, consequently reducing liver damage (Cerrah et al., 2023). Additionally, thymol administration has been noted to alleviate liver damage characterized by decreased ALT levels (Dou et al., 2022). The observed reduction in ALT due to the medicinal plants used in this study can be credited to their antioxidant, antimicrobial and anti‐inflammatory properties (Geyikoglu et al., 2019).

## 5 CONCLUSIONS

The findings from this study lead to the conclusion that both oregano and rosemary can mitigate the pathogenic impact of *C. jejuni*. These additives offer a viable alternative to *Erythromycin* in broiler feeding strategies. Nonetheless, employing higher concentrations is recommended if cinnamon is chosen for more robust outcomes. However, further experimentation is necessary to determine optimal cinnamon for achieving desired effects.

## AUTHOR CONTRIBUTIONS

**Zahra Alimohammadi**: Methodology; data curation; project administration. **Hassan Shirzadi**: Supervision; conceptualization; investigation; methodology; data curation; data analysis; funding acquisition; Writing — original draft. **Kamran Taherpour**: Conceptualization; investigation; methodology; data curation. **Enayat Rahmatnejad**: Investigation; methodology; writing; review and editing. **Ali Khatibjoo**: Investigation; methodology; validation; data analysis.

## CONFLICT OF INTEREST STATEMENT

The authors declare no conflicts of interest.

### ETHICS STATEMENT

All experimental protocols adhered to the guidelines, which were approved by the Animal Ethics Committee of Ilam University (Ilam, Iran) and were in accordance with the EU standards for the protection of animals and/or feed legislation. The ethical approval number based on the submitted MSc student thesis proposal is as follows: 9913/09, Anim Sci Dep.

### PEER REVIEW

The peer review history for this article is available at https://publons.com/publon/10.1002/vms3.70034.

## Data Availability

The data that support the findings of this study are available on the request of readers via the email address provided.
